# Does chocolate reduce blood pressure? A meta-analysis

**DOI:** 10.1186/1741-7015-8-39

**Published:** 2010-06-28

**Authors:** Karin Ried, Thomas Sullivan, Peter Fakler, Oliver R Frank, Nigel P Stocks

**Affiliations:** 1 Discipline of General Practice, The University of Adelaide, Adelaide, SA 5005, Australia; 2 Discipline of Public Health, The University of Adelaide, Adelaide, SA 5005, Australia

## Abstract

**Background:**

Dark chocolate and flavanol-rich cocoa products have attracted interest as an alternative treatment option for hypertension, a known risk factor for cardiovascular disease. Previous meta-analyses concluded that cocoa-rich foods may reduce blood pressure. Recently, several additional trials have been conducted with conflicting results. Our study summarises current evidence on the effect of flavanol-rich cocoa products on blood pressure in hypertensive and normotensive individuals.

**Methods:**

We searched Medline, Cochrane and international trial registries between 1955 and 2009 for randomised controlled trials investigating the effect of cocoa as food or drink compared with placebo on systolic and diastolic blood pressure (SBP/DBP) for a minimum duration of 2 weeks. We conducted random effects meta-analysis of all studies fitting the inclusion criteria, as well as subgroup analysis by baseline blood pressure (hypertensive/normotensive). Meta-regression analysis explored the association between type of treatment, dosage, duration or baseline blood pressure and blood pressure outcome. Statistical significance was set at *P *< 0.05.

**Results:**

Fifteen trial arms of 13 assessed studies met the inclusion criteria. Pooled meta-analysis of all trials revealed a significant blood pressure-reducing effect of cocoa-chocolate compared with control (mean BP change ± SE: SBP: -3.2 ± 1.9 mmHg, *P *= 0.001; DBP: -2.0 ± 1.3 mmHg, *P *= 0.003). However, subgroup meta-analysis was significant only for the hypertensive or prehypertensive subgroups (SBP: -5.0 ± 3.0 mmHg; *P *= 0.0009; DBP: -2.7 ± 2.2 mm Hg, *P *= 0.01), while BP was not significantly reduced in the normotensive subgroups (SBP: -1.6 ± 2.3 mmHg, *P *= 0.17; DBP: -1.3 ± 1.6 mmHg, *P *= 0.12). Nine trials used chocolate containing 50% to 70% cocoa compared with white chocolate or other cocoa-free controls, while six trials compared high- with low-flavanol cocoa products. Daily flavanol dosages ranged from 30 mg to 1000 mg in the active treatment groups, and interventions ran for 2 to 18 weeks. Meta-regression analysis found study design and type of control to be borderline significant but possibly indirect predictors for blood pressure outcome.

**Conclusion:**

Our meta-analysis suggests that dark chocolate is superior to placebo in reducing systolic hypertension or diastolic prehypertension. Flavanol-rich chocolate did not significantly reduce mean blood pressure below 140 mmHg systolic or 80 mmHg diastolic.

## Background

Flavanol-rich chocolate and cocoa products have attracted interest as nonpharmacological treatment options for high blood pressure, a known risk factor for cardiovascular disease [1,2]. Even small reductions in blood pressure substantially reduce cardiovascular risk [3,4]. Current guidelines strongly recommend integration of lifestyle modification and complementary treatment with the use of conventional blood pressure medications [5].

Polyphenols, in particular flavanols in cocoa products, have been shown to increase the formation of endothelial nitric oxide, which promotes vasodilation and consequently may lower blood pressure [6-8]. Meta-analyses by Taubert *et al*. (2007) [9], including five small trials [10-13], and Desch *et al*. (2010) [14], including 10 trials [10-13,15-20], concluded that cocoa-rich foods may reduce blood pressure. However, additional trials have been published since then, with conflicting results [21-24]. Our study updates current research on the effect of cocoa-rich products taken daily for two or more weeks compared with placebo on blood pressure in adults. In addition, we explored the influence of baseline blood pressure, dosage, duration, type of control, study design, age, body mass index and trial quality on blood pressure outcome.

## Methods

### Search strategy

We searched the Medline and Cochrane databases for randomised controlled trials of chocolate or cocoa on blood pressure published between 1955 and 2009 using the following search terms: chocolate OR cocoa AND blood pressure. We also searched reference lists of published studies and checked international trial registries http://www.clinicaltrials.gov; http://www.trialregister.nl; http://www.anzctr.org.au; http://www.controlled-trials.com for unpublished but completed studies investigating chocolate/cocoa for blood pressure.

### Selection of trials

Trials were included in the meta-analysis if the control group received a placebo or a low dose of flavanol-containing cocoa product (drink, bar or tablet), the trial duration was ≥ 14 days, and the clinical mean or median systolic or diastolic blood pressure (SBP/DBP) and standard deviation (SD) were available. We contacted authors of studies which did not report numerical mean SBP/DBP or SD and received datasets from two studies [18,22], which we included in the meta-analysis. Three eligible completed but unpublished studies were excluded because data were not available at the time of this study [25-27].

### Data extraction and quality assessment

Data were abstracted and quality was assessed independently by two investigators (KR, PF) using guidelines published by the Cochrane Collaboration [28] (Tables 1,2,3). Any disagreement was resolved by discussion between the authors (KR, PF) in consultation with the statistician (TS). Characteristics of trials included in the meta-regression analysis are shown in Table 1. We assessed quality on the basis of randomisation, blinding, whether blood pressure was a primary outcome measure, loss to follow-up, funding source and whether compliance and dietary chocolate intake had been assessed, as these could have influenced findings (Table 3). No trial was excluded in the meta-analysis on grounds of quality; however, higher-quality trials (score ≥ 3.5 of 5 points) were compared with lower-quality trials by meta-regression analysis.

**Table 1:** Characteristics of trials included in the meta-analysis

Trial; Location	Study design; treatment/control groups	Dosage; duration	Active ingredients per daily dosage	Mean age; mean BMI	N treatment/control	Mean SBP (SD) in mmHg at start/end of treatment versus control	Mean DBP (SD) in mmHg at start/end of treatment versus control
Taubert *et al*. [10]; Germany	Crossover^a,b^	100 g/d;	500 mg polyphenols	59.5 yr;	13/13	Cocoa: 153.4 (4.4)/148.6 (2.4);	Cocoa: 84.5 (4.6)/82.9 (4.6);
	Dark/white chocolate	14 d		24.1		Control: 153.6 (4.4)/154.0 (3.6)	Control: 84.2 (4.2)/84.5 (4.3)
Murphy et al. [15]; Australia	Parallel;	6 tablets/d	234 mg flavanols;	43.5 yr;	13/15	Cocoa: 118 (13)/120 (12);	Cocoa: 78 (12)/77 (10);
	High/low flavanol cocoa tablets	28 d	Control: 6.4 mg	26.0		Control: 116 (9)/119 (8)	Control: 76 (8)/76 (7)
Engler [11]; USA	Parallel;	46 g/d;	213 mg procyanidins	32.1 yr;	11/10	Cocoa: 121 (17.9)/120 (13.3)	Cocoa: 68.1 (8.3)/69 (6.6)
	High/low flavanoid chocolate	14 d		22.6		Control: 112.8 (8.9)/110 (6.3)	Control: 66.1 (5.4)/66 (6.3)
Fraga *et al*. [12]; USA	Crossover^a^;	105 g/d;	168 mg flavanols	18.0 yr;	27/28	Cocoa: 123 (11.2)/117 (7.5)	Cocoa: 72 (7.5)/67 (7.5)
	Dark/white chocolate	14 d		24.1		Control: 123 (11.2)/121 (7.5)	Control: 71 (7.5)/70 (7.5)
Grassi (normotensive arm) [13]; Italy	Crossover^a,b^;	100 g/d;	500 mg polyphenols	33.9 yr;	15/15	Cocoa: 112.9 (8.5)/105.9 (6.6)	Cocoa: 74.0 (5.7)/69.8 (4.5)
	Dark/white chocolate	15 d		22.6		Control: 113.2 (7.9)/112.7 (7.6)	Control: 73.8 (5.5)/73.5 (5.3)
Grassi *et al*. (hypertensive arm [13]; Italy	Crossover^a,b^;	100 g/d;	500 mg polyphenols	43.7 yr;	20/20	Cocoa: 141.3 (4.8)/129.3 (5.7);	Cocoa: 92.4 (3.8)/84.6 (5.6);
	Dark/white chocolate	15 d		25.4		Control: 141.1 (5.4)/140.4 (4.6)	Control: 91.8 (4.7)/91.2 (4.7)
Taubert *et al*. [16]; Germany	Parallel^a^;	6.3 g/d;	30 mg polyphenols	63.6 yr;	22/22	Cocoa: 147.7 (7.1)/144.8 (ng);	Cocoa: 86.4 (4.1)/84.5 (ng);
	Dark/white chocolate	126 d (18 wk)		24.0		Control: 147.5 (8.0)/147.6 (ng)	Control: 86.7 (3.8)/86.7 (n.g.)
Crews *et al*. [17]; USA	Parallel^a^;	37 g/d choc + 11 g/d drink;	754 mg proanthocyanins;	68.8 yr;	45/45	Cocoa: 126.8 (14.3)/123.3 (12.3);	Cocoa: 74.2 (8.2)/73.7 (7.5);
	Dark chocolate + cocoa drink/low flavanol chocolate + drink	42 d (6 wk)	Control: 41.1 mg	25.3		Control: 128.6 (14.3)/125.5 (12.7)	Control: 75.0 (8.0)/74.4 (7.7)
Grassi *et al*. [18]; Italy	Crossover^a,b^;	100 g/d;	1008 mg phenols	44.8 yr;	19/19	Cocoa: 141.1 (3.4)/137.3 (4.0);	Cocoa: 91.2 (4.2)/87.3 (4.6);
	Dark/white chocolate	15 d		26.5		Control: 140.9 (3.4)/140.8 (3.5)	Control: 91.1 (3.7)/90.9 (3.5)
Muniyappa *et al*. [20]; USA	Crossover^a,b^;	31 g/d cocoa;	900 mg polyphenols;	51.0 yr;	20/20	Cocoa: 141 (13)/139 (9);	Cocoa: 91 (13) 88 (9);
	High flavanol cocoa/low flavanol drink	14 d	Control: 28 mg	33.2		Control: 141 (13)/140 (9)	Control: 91 (13)/87 (9)
Davison *et al*. (nonexercise arm) [21]; Australia	Parallel;	300 ml drink mix/d;	902 mg flavanols;	44.9 yr;	12/11	Cocoa: 124 (10.4)/122.1 (ng);	Cocoa: 76 (6.2)/74.2 (ng);
	High flavanol cocoa/low flavanol drink	84 d (12 wk);	Control: 36 mg	33.6		Control: 124 (5.97)/128.2 (ng)	Control: 77 (5.0)/79.8 (ng)
Davison (exercise arm)[21]; Australia	Parallel;	300 ml drink mix/d;	902 mg flavanols;	45.4 yr;	13/13	Cocoa: 126 (10.4)/127.1 (ng);	Cocoa: 78 (8.7)/77.5 (ng);
	High flavanol cocoa/low flavanol drink	84 d (12 wk)	Control: 36 mg	33.4		Control: 121 (13.0)/120.5 (ng)	Control: 74 (5.8)/73.8 (ng)
Shiina *et al*. [22]; Japan	Parallel^a^;	45 g/d	550 mg polyphenols	29.8 yr;	20/19	Cocoa: 116.4 (12.7)/121.0 (12.7);	Cocoa: 64.7 (11.7)/71.3 (10.8);
	Dark/white chocolate	14 d		22.6		Control: 121.6 (14.9)/125.6 (11.4)	Control: 72.2 (13.8)/77.4 (11.6)
Ried *et al*. (phase 1) [23]; Australia	Parallel;	50 g/d;	750 mg polyphenols	53.1 yr;	11/10	Cocoa: 135.0 (12.5)/133.1 (11.7)^c^;	Cocoa: 83.6 (10.6)/84.5 (11.6);
	Dark chocolate/placebo pill	56 d (8 wk)		26.6		Control: 135.7 (12.4)/130.8 (18.3)^c^	Control: 77.8 (8.6)/77.3 (10.0)
Monagas *et al*. [24]; Spain	Crossover;	40g/d + 250 skim milk; control: 500 ml skim milk	495 mg polyphenols	69.7 yr;	42/42	Cocoa: 138 (26)/138 (16);	Cocoa: 84 (13)/82 (13);
	Cocoa powder in milk/milk only	28 d		27.6		Control: 138 (26)/135 (24)	Control: 84 (13)/81 (13)

^a^7-day cocoa/flavanol-free run-in period.

^b^7-day washout period before crossover.

^c^Median.

BMI, body mass index; DBP, diastolic blood pressure; g/d, grams per day; mg; milligrams; ml, millilitre; N, number of participants; ng, not given; SBP, systolic blood pressure; SD, standard deviation; yr, years; wk, weeks

**Table 2:** Characteristics of trials excluded from the meta-analysis

Trial; location	Study design; treatment/control groups	Dosage; duration	Active ingredients per daily dosage	Mean age; mean BMI	N treatment/control	Reason for exclusion
Grassi *et al*. [19]; Italy	Crossover^a,c^	100 g/d	500 mg polyphenols	33.9 yr;	15/15	Same population and protocol as studied in Grassi *et al*. [11]
	Dark/white chocolate	15 d		22.6		
Allen *et al*. [34], Erdman *et al*. [35]; USA	Crossover^b^;	22g/d	Not reported	44.7 yr;	44/44	No cocoa-free control group
	Chocolate + plant sterols/chocolate + no plant sterols	28 d (4 wk)		27.8		
Balzer *et al*. [36]; USA	Parallel;	3 drink mix/d	963 mg flavanols;	63.8 yr;	21/20	No mean SBP/DBP (SD) reported
	High/low-flavanol drink	30 d (4 wk)	control: 75 mg flavanols	32.1		
Faridi *et al*. [37]; USA	Crossover^a,c^;	74 g bar with 22 g cocoa/d;	821 mg flavanols	53 yr;	44/44	Duration < 2 wk
	Dark chocolate/placebo bar	Single dose, 1 d		30		
Ried *et al*. (phase 2) [23]; Australia	Crossover^d^;	50 g/d; control: 1 capsule/d;	750 mg polyphenols;	53.1 yr;	26/26	Crossover of two active treatment groups, no true control group
	Dark chocolate/Tomato extract capsules	56 days (8 wks)	control: tomato extract (15 mg lycopene)	26.6		
Almoosawi *et al*. [38]; UK	Crossover^a,c^;	20 g/d;	1000 mg polyphenols;	31 yr;	14/14	Two dosages, no true control group
	Dark chocolate dosage 1/dosage 2	14 days	control: 500 mg	27.7		

^a^7-day cocoa/flavanol-free run-in period.

^b^2-week run-in period.

^c^7-day washout period before crossover.

^d^4-week washout period.

For abbreviations, see Table 1 footnote.

**Table 3:** Quality assessment of trials included in the meta-analysis

Trial ID	Total score	Blinding	Outcome measure: blood pressure	Loss to follow-up	Funding source	Compliance; other chocolate diary
						
Scores given		1: choc + control blinded	1: Primary	1: < 20%	1: Sponsor not involved in data collection, analysis	1: Compl assessed; no sign diff between groups
		0.5: control blinded	0.5: Secondary	0: ≥ 20%	0: Sponsor involved	1: Compl assessed, sign diff included in analysis
		0: No blinding				0: Compl not assessed
						
Taubert *et al*. [10]	4	0: dark/white	1	1: none	1	1
Murphy *et al*. [15]	3.5	1: cocoa tablets	0.5	1: 9.5% (4 of 42)	0: supported by Mars	1: tablet count, 7 day weight food records x2
Engler *et al*. [11]	5	1: flav bar matched	1	1: none	1	1
Fraga *et al*. [12]	2.5	0: dark/white	1	1: 3.6% (1 of 28)	0: 3 authors from Mars	0.5: reported what consumed, no diary
Grassi *et al*. [13]	3.5	0: dark/white	1	1: none	0.5: unclear	1: daily diary
Grassi *et al*. [13]	3.5	0: dark/white	1	1: none	0.5: unclear	1: daily diary
Taubert *et al*. [16]	4	0: dark/white	1	1: none	1	1: food diary
Crews *et al*. [17]	2.5	0.5: flav drink; > 50% assumed correct group they were in	1	0.5: 11% (11 of 101)	0.5: independent industry research grant, supplier	0: not reported
Grassi *et al*. [18]	3.5	0: dark/white	1	1: none	0.5: unclear	1: diary
Muniyappa *et al*. [20]	3	1: flav drink; blinding assessed ok	1	0: 31% (9 of 29)	1	0: not reported
Davison *et al*. [21]	3	1: flav drink	1	0: 21% (14 of 65)	0: Mars Financial support	1: diet + background exercise
Davison *et al*. [21]	3	1: flav drink	1	0: 21% (14 of 65)	0: Mars Financial support	1: diet + background exercise
Shiina *et al*. [22]	2	0: dark/white	0	1: none	1	0
Ried *et al*. [23]	4.5	0.5: control blinded	1	1: 8% (3 of 39)	1	1: diary
Monagas *et al*. [24]	3.5	0: cocoa powder in milk/milk	0.5	1: none	1	1: diet monitoring (3-day food questionnaires x3)

All 15 trial arms were adequately randomised.

Choc, chocolate; compl, compliance; flav, flavanol; sign diff, significant difference;

### Analysis

Meta-analysis was conducted using the Cochrane Program Review Manager version 5 [29]. Owing to high heterogeneity between trials, we used a random effects model and considered subgroup meta-analysis by baseline mean blood pressure, similar to our recent meta-analysis of the effect of garlic on blood pressure [30]. For systolic blood pressure, trials were divided into a hypertensive subgroup (SBP ≥ 140 mmHg) and a normotensive subgroup (SBP < 140 mmHg) at the start of treatment. For diastolic blood pressure, a division into a higher BP subgroup (DBP ≥ 80 mmHg) and lower BP subgroup (DBP < 80 mmHg) at the start of treatment allowed an even distribution of trials between subgroups and reduction in heterogeneity.

Meta-regression analyses were conducted using Stata version 10 [31] to explore reasons for high heterogeneity in the pooled meta-analysis of all studies. The following variables were tested, as their associations with blood pressure outcomes are physiologically plausible: Dosage of polyphenols in the active treatment group (continuous variable), type of control (categorical variable: low-flavanol control as drink, tablet or bar/flavanol-free control as white chocolate, milk, or placebo capsules), duration (continuous and categorical > 2 weeks yes/no), study design (parallel versus crossover), starting SBP (continuous and categorical > 140 mmHg yes/no), starting DBP (continuous and categorical >80 mmHg yes/no), quality score (≥ 3.5 yes/no), average body mass index (BMI) (continuous and categorical > 25 or > 30 yes/no) and average age (continuous).

If meta-regression results indicated a variable to contribute significantly to heterogeneity between studies, subgroup analysis by this variable was conducted, testing whether there was an effect of treatment on blood pressure outcomes within each subgroup. If heterogeneity was reduced, the subgroup analysis provided a more reliable estimate of pooled effect size between the treatment groups. Additionally, sensitivity analysis excluding selected trials explored the robustness of results. Publication bias or small study effect was assessed by Begg's funnel plots and Egger's regression tests [32,33].

## Results

### Summary of included studies

**Figure 1:**
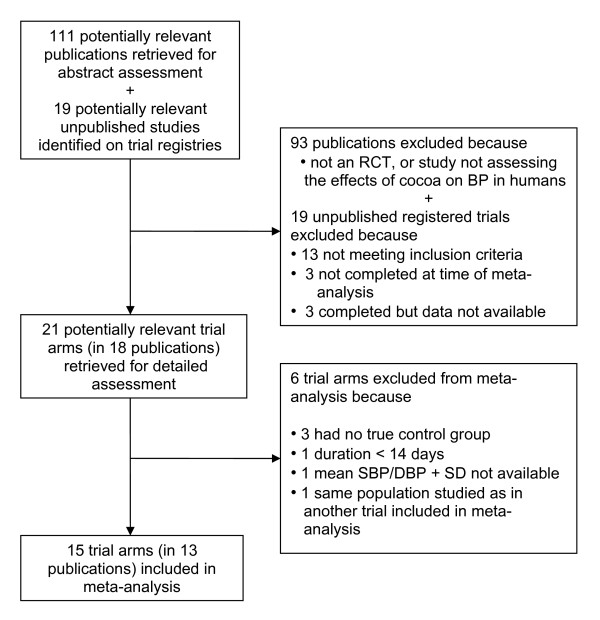
**Flow diagram of trial selection**.

A total of 18 publications including 21 trial arms were assessed in detail for inclusion [10-13,15-24,34-38] (Figure 1). Fifteen trial arms reported in 13 publications met the inclusion criteria [10-13,15-18,20-24] (Figure 1, Table 1). Six trial arms were excluded because 1) the same population and protocol were used in [19] compared with [13]; 2) the comparison group received other vasoactive substances rather than placebos as a) chocolate ± plant sterols [34,35], b) tomato extract in phase 2 of trial [23], or c) half dose of chocolate [38]; 3) mean SBP/DBP and SD were not reported and could not be obtained from the authors [36]; and 4) the trial was of 1-day duration [37] (Table 2).

The 15 trial arms included in our meta-analysis were adequately randomised; double-blinding was achieved in five trial arms using low-flavanol-containing products as control [11,15,20,21]. All but three trial arms [15,22,24] assessed blood pressure as the primary outcome measure, and 12 of the 15 trial arms had a participant attrition of less than 20% [10,11,13,15-18,22-24] (Table 3).

Eight trial arms used a parallel study design [11,15-17,21-23], and seven study arms used a crossover design [10,12,13,18,20,24]. All but two [12,24] of the seven crossover trials incorporated a washout period of 7 days between the alternate treatment period. In eight trials the intervention period was 2 weeks (14 or 15 days) [10-13,18,20,22], while longer intervention periods were trialled in seven studies (range 4-18 weeks) [15-17,21,23,24]. Eight trials employed a 7-day run-in period before commencing with the treatments [10,13,16-18,20,22] (Table 1).

Polyphenol content varied widely between the trials (range 30-1008 mg/day, Table 1). Although authors labelled the active ingredient in chocolate with a variety of terms (polyphenol, flavanol, proanthocyanidin, epicatechin and catechin), we are reasonably confident that the reported dosages of total polyphenol or measured derivates are comparable. There is some confusion in the literature about the correct labelling of the vasoactive chemical components in cocoa, and terms are often used interchangeably and sometimes incorrectly. Furthermore, the most commonly used methods for polyphenol content analysis (high performance liquid chromatography [39] and Folin-Ciocalteu method [40]) each measure both monomer (epicatechin and catechin) and oligomer (proanthocyanidin) polyphenol components. These polyphenol components belong to the flavanols or flavanoids, which make up 99% of all polyphenols in cocoa [41,42]. However, we were not able to compare findings by method of polyphenol content analysis, as details were not available for all trials.

We collated information on age and weight/BMI because age and BMI may influence responsiveness to blood pressure treatment [43,44]. Mean age and BMI varied substantially between trials: mean age ranged between 18 and 70 years, and mean BMI was in the overweight/obese category for 9 of the 15 trials (mean BMI: < 25, n = 6; 25-30, n = 6; > 30, n = 3).

### Meta-analysis

**Figure 2:**
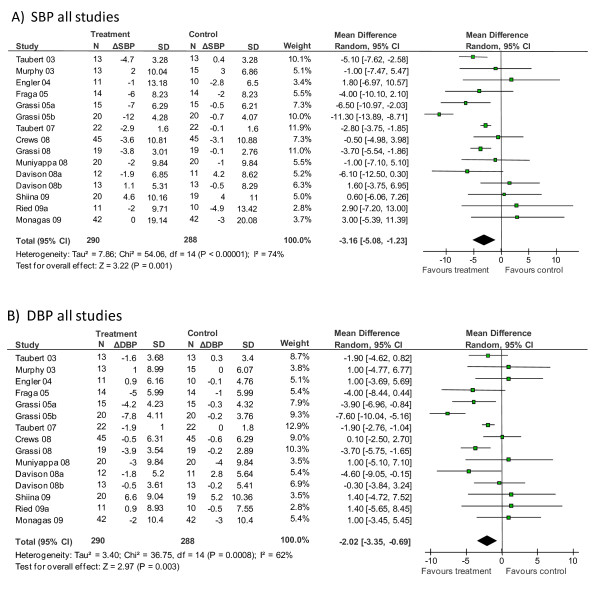
**Meta-analysis of the effect of chocolate/cocoa on (A) systolic blood pressure or (B) diastolic blood pressure**. N, number of participants; ΔSBP/ΔDBP, difference in mean SBP/DBP between start and end of intervention; SD, standard deviation; CI, confidence interval

Meta-analysis of all 15 trial arms revealed a significant blood pressure-reducing effect of cocoa/chocolate compared with control (pooled mean SBP: -3.16 [95% CI, -5.08, -1.23] mmHg, *P *= 0.001; pooled mean DBP: -2.02 [95% CI, -3.35, -0.69] mmHg, *P *= 0.003) (Figure 2). Heterogeneity between trials was high (SBP: *I *^2 ^= 74%; DBP: *I *^2 ^= 62%), prompting subgroup meta-analysis by baseline blood pressure as well as meta-regression and sensitivity analyses.

**Figure 3:**
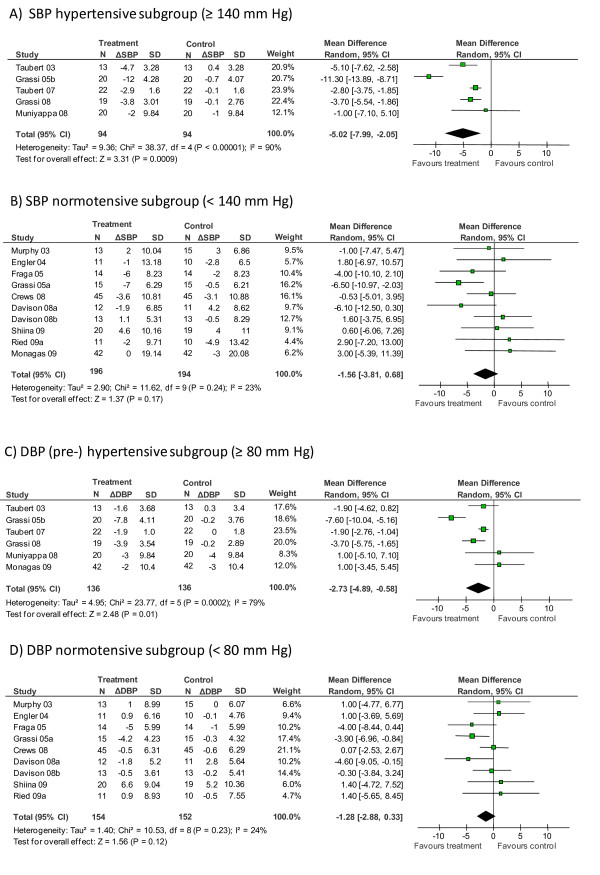
**Subgroup meta-analysis of the effect of chocolate/cocoa on (A) systolic blood pressure of hypertensive subjects (≥ 140 mmHg at baseline) or (B) 'normotensive' subjects (< 140 mmHg at baseline) and on (C) diastolic blood pressure of (pre-)hypertensive subjects (≥ 80 mmHg) or (D) 'normotensive' subjects (< 80 mmHg)**. See Figure 2 legend for abbreviation definitions.

We pooled trial arms with mean baseline SBP in the hypertensive range (SBP ≥ 140 mmHg) and trial arms with mean baseline SBP of < 140 mm Hg. While meta-analysis of the SBP hypertensive subgroup remained significant (SBP_hyper_: -5.02 [95% CI, -7.99, -2.05] mmHg; *P *= 0.0009; Figure 3A), meta-analysis of the SBP normotensive subgroup demonstrated no significant difference in blood pressure reduction between the chocolate/cocoa group and the control group (SBP_normo_: -1.56 [95% CI, -3.81, 0.68] mmHg, *P *= 0.17; Figure 3B). Heterogeneity remained high in the hypertensive subgroup, but was reduced in the SBP normotensive subgroup (SBP_hyper_: *I *^2 ^= 90%; SBP_normo_: *I *^2 ^= 23%).

For subgroup analysis of diastolic blood pressure, we pooled trial arms with mean baseline DBP in the hypertensive and prehypertensive range (DBP ≥ 80 mmHg) and trials with mean baseline DBP < 80 mm Hg. Three of the six trial arms in the subgroup with DBP ≥ 80 mmHg reported mean DBP values at baseline in the hypertensive range (≥ 90 mmHg) [13,18,20], while three trials reported mean DBP at baseline in the prehypertensive range (84-86 mmHg) [10,16,24].

Results of DBP subgroup analyses were similar to the results of SBP subgroup analyses. While the DBP pre-/hypertensive subgroup analysis remained significant (DBP_hyper_: -2.73 [95% CI, -4.89, -0.58] mmHg, *P *= 0.01; *I *^2 ^= 79%; Figure 3C), DBP normotensive subgroup analysis demonstrated no significant difference between chocolate and control groups (DBP_normo_: -1.28 [95% CI, -2.88, 0.33] mmHg, *P *= 0.12; *I *^2 ^= 24%; Figure 3D).

**Figure 4:**
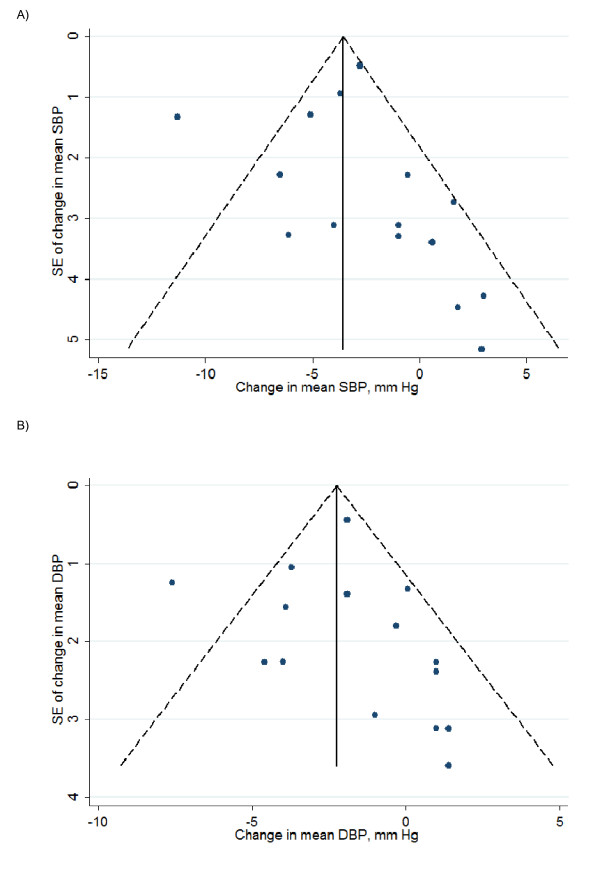
**Funnel plots of trials included in the meta-analysis for (A) systolic blood pressure and (B) diastolic blood pressure**. The vertical line of Begg's funnel plot represents the pooled mean effect size, and the dotted lines represent the 95% confidence interval. *P *values are derived from Egger's test.

Funnel plots and Egger's test indicated no publication bias (Figure 4).

### Meta-regression and sensitivity analysis

Five variables tested (dosage, duration, quality score, age and BMI) did not show any significant association with blood pressure outcomes in the meta-regression analysis, while study design (parallel versus crossover) was borderline significantly associated with BP outcome (SBP: *P *= 0.053; DBP: *P *= 0.060); and type of control (flavanol-free control versus low-flavanol product as control) was borderline significant for DBP (*P *= 0.052).

Subgroup analyses revealed reduced heterogeneity in the subgroup including parallel trials, but high heterogeneity remained in the subgroup with crossover trials (Table 4). Similarly, the subgroup including low-flavanol products as control demonstrated lower heterogeneity and no significant difference between treatment groups, in contrast to the subgroup with flavanol-free unblinded controls (Table 4).

**Table 4:** Subgroup analyses by study design and type of control

Subgroups		SBP			DBP		
		Mean difference in mmHg (95% CI)	*P*	***I***^**2**^	Mean difference in mmHg (95% CI)	*P*	***I***^**2**^
Study design	Crossover [10,12,13,18,20,21,23]	-4.91 (-7.94, -1.88)	0.002	80%	-3.24 (-5.36, -1.12)	0.003	67%
	Parallel [11,15-17,21-23]	-2.01 (-3.42, -0.60)	0.005	9%	-1.20 (-2.29, -0.11)	0.03	12%
	Parallel^a ^[11,15,17,21-23]	-0.43 (-2.80, 1.94)	0.72	0%	-0.25 (-1.83, 1.33)	0.75	0%
Type of control	Flavanol-free: white chocolate, milk, pills [11,15,17,20,21]	-4.21 (-6.65, -1.77)	0.0007	82%	-2.81 (-4.52, -1.10)	0.001	70%
	Low-flavanol: drink, bar, tablet [10,13,16,18,22-24]	0.74 (-2.92, 4.40)	0.69	53%	-0.38 (-2.00, 1.24)	0.65	0%

SBP, systolic blood pressure; DBP, diastolic blood pressure; mmHg, millimetre mercury; CI, confidence interval; *P*, probability value (*P *< 0.05 statistically significant); *I*^2^, heterogeneity test.

^a^Sensitivity analysis excluding trial [16], as this trial used a notably lower dose and longer duration than the other trials included in the meta-analysis.

However, the results of these subgroup analyses need to be interpreted cautiously, as three of seven trial arms with a crossover study design were conducted by the same study team [13,18], and five of nine study arms using a flavanol-free control were conducted by two teams within similar population groups [10,13,16,18]. Therefore, study design or type of control might in fact be indirect predictors of BP outcome through other factors such as dietary habits of the study population.

Sensitivity analyses excluding trial [16], as this trial used a notably lower dose and longer duration than the other trials, did not change the results appreciably, with one exception: A small but statistically significant difference between treatment groups in subgroup analyses of trials with a parallel design shifted to a nonsignificant effect when trial [16] was excluded (Table 4).

## Discussion

Our meta-analysis including 15 trial arms demonstrated a small but significant blood pressure-reducing effect of flavanol-rich cocoa products compared with control (mean BP change ± SE: SBP: -3.2 ± 1.9 mmHg, *P *= 0.001; DBP: -2.0 ± 1.3 mmHg, *P *= 0.003). These effect sizes were smaller than in previous meta-analyses including fewer trials (Taubert *et al*. (2007), 5 trials, SBP: -4.7 ± 2.9 mmHg, *P *= 0.002; DBP: -2.8 ± 2.0 mmHg, *P *= 0.006, [9]; and Desch *et al*. (2010), 10 trials, SBP: -4.5 ± 1.4 mmHg, *P *= 0.001, DBP: -2.5 ± 1.4 mmHg, *P *= 0.001, [14]).

In contrast to previous meta-analyses [9,14], subgroup analyses in our larger meta-analysis suggested that there is a difference in outcome dependent on baseline blood pressure (hypertensive versus normotensive). While meta-analyses of the hypertensive subgroups found significant reductions (SBP: -5.0 ± 3.0 mmHg, *P *= 0.0009; DBP: -2.7 ± 2.2 mmHg, *P *= 0.01), analyses of normotensive subgroups did not demonstrate a significant reduction in blood pressure of flavanol-rich cocoa products (SBP: -1.6 ± 2.3 mmHg, *P *= 0.17; DBP: -1.3 ± 1.6 mmHg, *P *= 0.12). These findings are in line with meta-analyses of other nutritional supplements on blood pressure, which similarly found that blood pressure was significantly reduced in hypertensive subgroups but not in the normotensive subgroups [30,45].

Heterogeneity was reduced satisfactorily in the normotensive subgroups, indicating that trials in these subgroup analyses are highly comparable and the meta-analyses results can be interpreted with confidence. In contrast, heterogeneity remained high in the hypertensive subgroups, influenced greatly by one relatively small study arm [13], which demonstrated a large blood pressure reduction not matched by the other trials. Therefore, effect sizes and levels of significance of the subgroup meta-analyses of trials with (pre-)hypertensive subjects at baseline should be interpreted more cautiously.

The relatively modest but significant blood pressure-lowering effect of cocoa in the hypertensive subgroup is clinically relevant: a decline of 5 mmHg in systolic blood pressure may reduce the risk of a cardiovascular event by about 20% over 5 years [46]. Furthermore, the effect of cocoa in a hypertensive population is comparable to other lifestyle modifications, such as moderate physical activity (30 min/d) may reduce SBP by 4-9 mmHg [5].

Meta-regression analyses suggested study design (parallel versus crossover) and type of control (flavanol-free versus low-flavanol) to be significant predictors of blood pressure outcome but failed to show any statistically significant associations in the other variables tested. However, study design as well as type of control might be indirect predictors, as about half the trials using a crossover design and white chocolate as flavanol-free control were conducted by the same two teams and within similar study populations [10,13,16,18]. It is possible that participants shared characteristics that contributed to their responsiveness to cocoa products, such as local dietary habits or genetic/ethnic disparity [47,48]. Inclusion of trial location as a variable was impractical in our meta-regression analysis; however, future research may explore this further.

Furthermore, results of trials using flavanol-free controls, including white chocolate or milk, might overestimate the effect of the active treatment, owing to potential bias of unblinded participants. Therefore, related subgroup analyses need to be interpreted cautiously.

Meta-regression analysis did not suggest an association between dosage, duration, quality of trials, age, BMI and blood pressure outcome. However, inclusion of future trials in meta-regression analysis might provide further insight into predicting factors.

While regular consumption of flavanol-rich cocoa products may have a beneficial short-term effect in reducing blood pressure in hypertensive individuals, the practicability of chocolate or cocoa drinks as long-term treatment is questionable. A recent small study by our team investigating the acceptability of commercially available chocolate bars as an alternative treatment to capsules concluded that daily chocolate consumption for blood pressure may not be an acceptable and practical treatment option [23].

## Conclusion

Our meta-analysis of 15 trial arms suggests that dark chocolate and flavanol-rich cocoa products are superior to placebo in reducing systolic hypertension and diastolic prehypertension. However, flavanol-rich cocoa products did not significantly reduce mean blood pressure below 140 mmHg systolic or 80 mmHg diastolic. Additional trials of hypertensive populations are needed to elucidate whether local dietary habits or genetic factors influence the blood pressure-lowering effect of cocoa.

## List of Abbreviations

BP: blood pressure; BMI: body mass index; CI: confidence interval; DBP: diastolic blood pressure; mg: milligrams; mm Hg: millimetre mercury; RCT: randomised controlled trial; SBP: systolic blood pressure; SD: standard deviation.

## Competing interests

The authors declare that they have no competing interests.

## Authors' contributions

KR, ORF and NPS conceptualised the study and obtained funding. Data was acquired independently by KR and PF. KR and TS undertook data analysis and interpretation. KR prepared the manuscript with contributions from all co-authors. All authors approved the final version.

## Pre-publication history

The pre-publication history for this paper can be accessed here:

http://www.biomedcentral.com/1741-7015/8/39/prepub
